# HPM-Based Dynamic Sparse Grid Approach for Perona-Malik Equation

**DOI:** 10.1155/2014/417486

**Published:** 2014-06-23

**Authors:** Shu-Li Mei, De-Hai Zhu

**Affiliations:** ^1^College of Information and Electrical Engineering, China Agricultural University, Beijing 100083, China; ^2^Key Laboratory of Agricultural Information Acquisition Technology, Ministry of Agriculture, China Agricultural University, Beijing 100083, China

## Abstract

The Perona-Malik equation is a famous image edge-preserved denoising model, which is represented as a nonlinear 2-dimension partial differential equation. Based on the homotopy perturbation method (HPM) and the multiscale interpolation theory, a dynamic sparse grid method for Perona-Malik was constructed in this paper. Compared with the traditional multiscale numerical techniques, the proposed method is independent of the basis function. In this method, a dynamic choice scheme of external grid points is proposed to eliminate the artifacts introduced by the partitioning technique. In order to decrease the calculation amount introduced by the change of the external grid points, the Newton interpolation technique is employed instead of the traditional Lagrange interpolation operator, and the condition number of the discretized matrix different equations is taken into account of the choice of the external grid points. Using the new numerical scheme, the time complexity of the sparse grid method for the image denoising is decreased to* O*(4^*J*+2*j*^) from* O*(4^3*J*^), (*j* ≪ *J*). The experiment results show that the dynamic choice scheme of the external gird points can eliminate the boundary effect effectively and the efficiency can also be improved greatly comparing with the classical interval wavelets numerical methods.

## 1. Introduction

The nonlinear difference equation has been widely used in various fields in the past few decades such as the option pricing [1], stochastic analysis [2], hydrodynamics [3], and image processing [4]. Many powerful and efficient methods to find analytic solutions of nonlinear equation have drawn a lot of interest by a diverse group of scientists. These methods include the tanh-function method, the extended tanh-function method [5, 6], the sine-cosine method [7], the variational iteration method [8, 9], the homotopy perturbation method [10, 11], and Exp-function method [12].

As an excellent medical image processing model, the Perona-Malik model [4] has been widely used in image denoising in recent years. Perona-Malik model is a nonlinear 2-dimension partial differential equation in itself, which overcomes the drawback of the scale-space technique introduced by Witkin which involves generating coarser resolution images by convolving the original image with a Gaussian kernel. In this approach, a new definition of scale-space was suggested, and a class of algorithms was introduced; then accurately the locations of the “semantically meaningful” edges at coarse scales using a diffusion process can be obtained; that is, a high quality edge detector which successfully exploits global information was obtained with this new method.

It is very difficult to find the exact analytical solution of the Perona-Malik model as it is a nonlinear partial differential equation. Conventional methods for numerical solutions of partial differential equations mostly fall into three classes: finite difference methods, finite element methods, and spectral methods. Briefly, the finite difference method consists in defining the different unknowns by their values on a discrete grid and in replacing differential operators by difference operators using neighboring points. In the finite element method, the equations are integrated against a set of linear independent test functions with small compact support, and the solution is considered as a linear combination of this set of test functions. In spectral methods, the unknown functions are developed along a basis of functions having global support. This development is truncated to a finite number of terms which satisfy a system of coupled ordinary differential equations in time. The advantage of using either of the first two numerical techniques is the simplicity in adapting to complex geometries, while the main advantage of spectral methods is the greater accuracy [13, 14].

If the solution of a partial differential equation is regular, any of the three above-mentioned numerical techniques can be applied successfully. It is obvious that most of the images are irregular. This makes the Perona-Malik equation particularly difficult to resolve numerically using the above-mentioned methods. Spectral methods are not easily implemented because the irregularity of the solution causes the loss of high accuracy. Moreover, the global support of the basis function induces the well-known Gibbs phenomenon, which appears as the artifacts in images. Wavelet analysis is a new numerical concept which allows one to represent a function in terms of a set of basis function, called wavelets, which are localized both in location and in scale. Up to now, the finite difference method is the primary numerical algorithm for Perona-Malik model, which can bring artifact into the images due to the nonsmoothness of the basis function of the finite difference method [15, 16] as has been said before. The multilevel wavelet numerical method for the nonlinear PDEs has been proposed over ten years, which can take full advantage of the adaptability of the wavelet analysis [17]. The artifacts in image can be eliminated with the wavelet numerical algorithm instead of the finite difference method, as wavelet basis function possesses many excellent properties such as smoothness and compact support. But the support range of wavelet function is much wider than the basis function in the finite difference method [18, 19]. This leads to a lower computational efficiency of wavelet transform in solving 2D nonlinear PDEs. Besides, most of the wavelet algorithms for solving partial differential equations can handle periodic boundary conditions easily. The treatment of general boundary conditions is still an open question especially in solving the nonlinear problems. Construction of the wavelet defined in the interval (interval wavelet) is another good choice to handle the boundary conditions [20, 21]. Compared to the interpolation wavelet, a linear mapping between the external collocation points and the interval ones was supplied in the interval wavelet. The choice of the external collocation points depends on the smoothness and the gradient near each collocation point of the solution of the PDEs. Besides, the condition number of the system of equations obtained by the wavelet collocation method should be taken into account.

To an image with 2^*J*^∗2^*J*^ pixels (*J* ∈ *Z*), the Perona-Malik equation can be discretized into a system of ODEs with 4^*J*^-dimension by the coupling technique of HPM [22–27] and the wavelet collocation method [28, 29]. The corresponding time complexity is about *O*(4^3*J*^) with the variational iterative method for the system of ODEs [30]. Obviously, it does not meet the requirement of the larger image processing. Partitioning technique is the effective measure to improve the efficiency of this problem. In other words, the image should be divided into several blocks before denoising to the images. In each of image blocks, the multiple programs can be executed simultaneously. This is similar to the finite element method to some extent. Obviously, if the size of the image blocks is adaptive to whole image, the algorithm efficiency can be improved furthermore. Our research focuses on the general frame of sparse grids and the dynamic choice scheme of the external grid points, which can be used to decrease the boundary effect of each image block, and so, we just talk about the even partitioning in this paper for simplification.

The sparse representation of functions via a linear combination of a small number of basic functions has recently received a lot of attention in several mathematical fields such as approximation theory as well as signal and image processing. The advantage of the sparse grid approach is that it can be extended to nonsmooth solutions by adaptive refinement methods; that is, it can capture the steep waves that appeared in the solution of the PDEs. The main objective of the paper is to present a dynamic choice scheme of the external grid points and a general sparse grid operator for solving the Perona-Malik equation. In other words, the dynamic sparse grid approach provides an adaptive choice scheme on both of the external and the internal grid points. In the presentation of the method, we try to be as general as possible, giving only the main philosophy of the method and leaving some freedom for further exploration of its applications. Both the boundary condition and the condition number are addressed in this work. The first is how to incorporate the dynamic choice scheme on external grid points with the interpolation wavelet basis to construct an effective algorithm of solving partial differential equation. The second is how to construct a stable, accurate, and efficient numerical algorithm for the image denoising model.

## 2. Construction of Dynamic Sparse Grid Operator

There are many ways to eliminate the boundary effect from the multiscale basis. A simple solution is the even 2-periodical extension $\tilde{f}$ of function *f* : [0,1] → *R*, which is usually used in image analysis. Unfortunately, this extension generally produces discontinuities at the integers that are indicated by the large transform coefficients near the endpoints 0 and 1. Thus the constructed multiscale basis cannot exactly analyze the boundary behavior of a given function. To solve this problem, the popular method is using special boundary and interior scaling functions such as the interval wavelet to reduce the numerical problem at the boundaries. To the interpolation basis function, the common approach is to define the interpolation basis in the interval with the Lagrange multiplier. In fact, the Lagrange multiplier can be viewed as a map operator, which maps the external collocation points into the definition domain in the multiscale interpolation method. The choice of the amount of the external points relates to the smoothness and gradient near the boundary of the approximated function. In addition, another factor that we should take into account is the condition number of the system of ODEs obtained by the multiscale numerical method.

Obviously, the amount of the external collocation points should be different to different boundary conditions such as the smoothness, gradient near the boundary, and the condition number. In the partition technique about the image processing, the boundary conditions of the different image blocks are obviously different as the randomness of the image. In the representation, we try to give a dynamic choice scheme about the external collocation points to meet the requirement of the image partition technique, in which all above 3 factors are taken into account.

In the presentation of the method, we try to be as general as possible, giving only the main philosophy of the method and leaving some freedom for further exploration of its applications. We illustrate the method using two classical interpolation wavelets: Shannon wavelet and the autocorrelation function of Daubechies scaling functions. But we do not try to predict what wavelet is the best for our algorithm (it is simply impossible, due to the fact that some wavelets work better for some problems and worse for others).

### 2.1. Basis Functions with Interpolation Property

There are many wavelet functions which possess the interpolation property. The familiar interpolation wavelets family includes Shannon wavelet, Haar wavelet, and Faber-Schauder. Furthermore, it is easy to understand that the autocorrelation function of the orthogonal wavelet function also has the interpolation property. So, the autocorrelation function of the Daubechies scaling function is often employed to construct the wavelet collocation method.

The representation of Shannon wavelet [31, 32] is based upon approximating the Dirac delta function as a band-limited function and is given by

(1) $$\begin{array}{c} \phi \left(x\right)=\frac{\sin\left(\pi x\right)}{\pi x}. \end{array}$$

The Shannon wavelet possesses many excellent numerical properties such as interpolating, relative sparse, and orthogonal properties. A perceived disadvantage of (1) is that it tends to zero quite slowly as |*x*| → *∞*. A direct consequence of this is that there are a large number of grid points will contribute to the derivatives calculation of approximated function. For this reason Hoffman et al. [33] have suggested using the Shannon-Gabor wavelet as follows:

(2) $$\begin{array}{c} w\left(x\right)=\frac{\sin\left(\pi x\right)}{\pi x}\exp\left(-\frac{x^{2}}{2\sigma^{2}}\right),\;\sigma >0, \end{array}$$

where *σ* is the width parameter (or called window size). It has been proofed that (2) can improve the localized and asymptotic behavior of the Shannon scaling function. A consequence of this is that it ensures that derivatives at any one point are more dependent on the neighboring nodal values than on the nodal values further away from the point considered. However, the presence of the Gaussian window destroys the orthogonal properties possessed by the Shannon wavelet, effectively worsening the approximation to a Dirac delta function. In the following, the Shannon wavelet representation of the Dirac delta function is adopted, and it is shown that this representation ensures that the approach is identical to the weighted residual approach.

The autocorrelation functions of compactly supported scaling functions were first studied in the context of the Lagrange iterative interpolation scheme in [34]. Let *ϕ*(*x*) be the autocorrelation function:

(3) $$\begin{array}{c} \phi \left(x\right)=\overset{\infty }{\underset{-\infty }{\int }}\varphi \left(y\right)\varphi \left(y-x\right)dy, \end{array}$$

where *φ*(*x*) is the scaling function which appears in the construction of compactly supported wavelet. The function *ϕ*(*x*) is exactly the “fundamental function” of the symmetric iterative interpolation scheme introduced in [35]. Thus, there is a simple one-to-one correspondence between iterative interpolation schemes and compactly supported wavelet. In particular, the scaling function corresponding to Daubechies's wavelet with two vanishing moments yields the scheme in [36]. In general, the scaling functions corresponding to Daubechies's wavelets with *M* vanishing moments lead to the iterative interpolation schemes which use the Lagrange polynomials of degree 2*M*. Additional variants of iterative interpolation schemes may be obtained using compactly supported wavelets described in [37].

### 2.2. Construction of Dynamic Interpolation Wavelet in Interval

According to the definition of the interval wavelet, the interval interpolation basis functions can be expressed as

(4) $$\begin{array}{c} w_{jk}^{}\left(x\right)=\{\begin{array}{cc} \phi \left(2^{j}x-k\right)+\overset{-1}{\underset{n=\;-L+1}{\sum }}a_{nk}\phi \left(2^{j}x-n\right), & \\ \;k=0,\ldots ,L & \\ \phi \left(2^{j}x-k\right), & \\ \;k=L+1,\ldots ,2^{j}-L-1 & \\ \phi \left(2^{j}x-k\right)+\overset{2^{j}+L-1}{\underset{n=2^{j}+1}{\sum }}b_{nk}\phi \left(2^{j}x-n\right), & \\ \;k=2^{j}-L,\ldots ,2^{j}, & \end{array} \end{array}$$

where

(5) $$\begin{array}{c} a_{nk}=\overset{-1}{\underset{\begin{array}{c} i=L-1\\ i\neq k \end{array}}{\prod }}\frac{x_{j,n}-x_{j,i}}{x_{j,k}-x_{j,i}},\;\;b_{nk}=\overset{2^{j}+1+L}{\underset{\begin{array}{c} i=2^{j}+1\\ i\neq k \end{array}}{\prod }}\frac{x_{j,n}-x_{j,i}}{x_{j,k}-x_{j,i}}\\ x_{j,k}=k\frac{x_{\max}-x_{\min}}{2^{j}},\;k\in Z, \end{array}$$

where *L* is the amount of the external collocation points; the amount of discrete points in the definition domain is 2^*j*^ + 1  (*j* ∈ *Z*)*;* and [*x*_min⁡_, *x*_max⁡_] is the definition domain of the approximated function. Equations (4) and (5) illustrate that the interval wavelet is derived from the domain extension. The supplementary discrete points in the extended domain are called external points. The value of the approximated function at the external points can be obtained by Lagrange extrapolation method. Using the interval wavelet to approximate a function, the boundary effect can be left in the supplementary domain; that is, the boundary effect is eliminated in the definition domain.

According to (4) and (5), the interval wavelet approximant of the function *f*(*x*)   *x* ∈ [*x*_min⁡_, *x*_max⁡_] can be expressed as

(6) fj(x)=∑fj(xn)wj(2jx−n),xn=xmin⁡+nxmax⁡−xmin⁡2j,

where *f*_*j*_(*x*_*n*_) is the given value at the discrete point *x*_*n*_. At the external points, *f*_*j*_(*x*_*n*_) can be obtained by extrapolation; that is,

(7) fj(xn)={∑k=0L−1(fj(xk)∏i=0i≠kL−1xn−xixk−xi), n=−1,…,−L∑k=2j−L+12j(fj(xk)∏i=2j−L+1k≠i2jxn−xixk−xi), n=2j+1,…,2j+L.

So, the interval wavelet approximant of *f*(*x*) can be rewritten as

(8) fj(x)=∑n=−L−1(∑k=0L−1fj(xk)∏i=0L−1xn−xixk−xi)ω(2jx−n) +∑n=02jfj(xk)ω(2jx−n) +∑n=2j+12j+L(∑k=2j−L2jfj(xk)∏i=2j−L2jxn−xixk−xi)ω(2jx−n).

Let

(9) LSL(xn)=  ∑k=0L−1fj(xk)∏i=0L−1xn−xixk−xi,LEL(xn)=  ∑k=2j−L2jfj(xk)∏i=2j−L2jxn−xixk−xi.

Then,

(10) fj(x)=∑n=−L−1LSL(xn)ω(2jx−n)+∑n=02jfj(xk)ω(2jx−n) +∑n=2j+12j+LLEL(xn)ω(2jx−n),

where *LS*_*L*_(*x*_*n*_) and *LE*_*L*_(*x*_*n*_) correspond to the left and the right external points, respectively. They are obtained by Lagrange extrapolation using the internal collocation points near the boundary. So, the interval wavelet's influence on the boundary effect can be attributed to Lagrange extrapolation. It should be pointed out that we did not care about the reliability of the extrapolation. The only function of the extrapolation is enlarging the definition domain of the given function which can avoid the boundary effect that occurred in the domain. Therefore, we can discuss the choice of *L* by means of Lagrange inner- and extrapolation error polynomial as follows:

(11) RL(x)=f(L+1)(ξ)(L+1)!∏i=0L(x−xi),for  some  ξ  between  x,x0,…,xL.

Equation (11) indicates that the approximation error is related to both the smoothness and the gradient of the original function near the boundary. Setting different *L* can satisfy the error tolerance.

### 2.3. Adaptive Interval Interpolation Wavelet

The interval interpolation wavelet is often used to solve the diffusion PDEs with Neumann boundary conditions. The smoothness and gradient of the PDE's solution usually vary with the time parameter. If the parameter *L* is a constant, we have to take a bigger value in order to obtain result with higher calculation precision. But the bigger *L* usually introduces the famous Gibbs phenomenon into the numerical solution, which usually makes the algorithm become invalid. In addition, the bigger *L* will bring much more calculation. To keep higher numerical precision and save calculation, the best way is to design a procedure that *L* can vary with the curve's smoothness and gradient dynamically.

In this dynamic procedure, the error estimation equation (11) can be taken as the criterion about *L*. But in most cases, we cannot know the smoothness and the derivative's order of the original function. This can be solved by substituting the difference coefficient for the derivative. This is coincident with the Newton interpolation equation which is equivalent with Lagrange interpolation equation. In addition, the Lagrange interpolation algorithm has no inheritance which is the key feature of Newton interpolation. So, the basis function has to be calculated repeatedly as interpolation points are added into the calculation, which increases the computation complexity greatly. In contrast to the Lagrange method, the advantage of Newton interpolation method is that the basis function need not be recalculated as one point is added except only one more term which is needed to be added, which reduces the number of computed operations, especially the multiplication. So, it is convenient using the Newton interpolation method to construct the dynamic procedure.

#### 2.3.1. Newton Interpolation

The expression of Newton interpolation can be written as

(12) Nn(x)=f(x0)+(x−x0)f(x0,x1) +(x−x0)(x−x1)f(x0,x1,x2)+⋯ +(x−x0)(x−x1)⋯(x−xn−1) ×f(x0,x1,…,xn).

Substitute the Newton interpolation instead of the Lagrange interpolation into (29), which can be rewritten as

(13) fj(x)=∑n=−L−1(NSL(xn))ω(2jx−n) +∑n=02jfj(xn)ω(2jx−n) +∑n=2j+12j+L(NEL(xn))ω(2jx−n),

where

(14) NSL(xn)=f(x0)+(xn−x0)f(x0,x1) +(xn−x0)(xn−x1)f(x0,x1,x2)+⋯ +(xn−x0)(xn−x1)⋯(xn−xL−1) ×f(x0,x1,…,xL),NSR(xn)=f(x2j)+(xn−x2j)f(x2j,x2j−1) +(xn−x2j)(xn−x2j−1)f(x2j,x2j−1,x2j−2) +⋯+(xn−x2j) ×(xn−x2j−1)⋯(xn−x2j−L) ×f(x2j,x2j−1,…,x2j−L).

#### 2.3.2. Relation between the Newton Interpolation Error and the Choice of *L*

It is well known that the Newton interpolation is equivalent to the Lagrange interpolation. The corresponding error estimation can be expressed as

(15) $$\begin{array}{c} R_{n}\left(x\right)=\left(x-x_{0}\right)\left(x-x_{1}\right)\cdots \left(x-x_{n}\right)f\left(x,x_{0},\ldots ,x_{n}\right). \end{array}$$

And the simplest criterion to terminate the dynamic choice on *L* is |*R*_*n*_(*x*)| ≤ *T*_*a*_ (*T*_*a*_ is the absolute error tolerance). Obviously, it is difficult to define *T*_*a*_ which should meet the precision requirement of all approximated curves. In fact, the difference coefficient *f*(*x*, *x*_0_, …, *x*_*n*_) can be used directly as the criterion; that is,

(16) $$\begin{array}{c} \left|f\left({x,x}_{0},\ldots ,x_{n}\right)\right|<\;\varepsilon . \end{array}$$

As mentioned above, once the curves with lower order smoothness are approximated by higher order polynomial expression, the errors will become bigger on the contrary. In fact, even if the *L* is infinite, the computational precision cannot be satisfied except increasing computational complexity. To avoid this, we design the termination procedure of dynamic choice about *L* as follows:

 if *f*(*x*_0_, *x*_1_) < *T*_*a*_, then *L* = 1,
 else, if *f*(*x*_0_, *x*_1_, *x*_2_) < *T*_*a*_ or *f*(*x*_0_, *x*_1_, *x*_2_) < *f*(*x*_0_, *x*_1_), then *L* = 2,
 else, if *f*(*x*_0_, *x*_1_, *x*_2_, *x*_3_) < *T*_*a*_ or *f*(*x*_0_, *x*_1_, *x*_2_, *x*_3_) < *f*(*x*_0_, *x*_1_, *x*_2_), then *L* = 3,

#### 2.3.3. *L* and the Condition Number of the System of Algebraic Equations

In the field of numerical analysis, the condition number of a function with respect to an argument measures how much the output value of the function can change for a small change in the input argument. This is used to measure how sensitive a function is to changes or errors in the input and how many errors in the output result from an error in the input. There is no doubt that the choice of *L* can change the condition number of the system of algebraic equations discretized by the wavelet interpolation operator or the finite difference method. Therefore, the choice of *L* should take the condition number into account. In fact, if the condition number cond(*A*) = 10^*k*^, then you may lose up to *k* digits of accuracy on top of what would be lost to the numerical method due to loss of precision from arithmetic methods [34]. According to the general rule of thumb, the choice should follow the rule as follows:

(17) $$\begin{array}{c} \frac{\text{Cond}\left(A_{L+1}\right)}{\text{Cond}\left(A_{L}\right)}<10. \end{array}$$

#### 2.3.4. Relation between *L* and Computation Complexity

The computational complexity of interpolation calculation is not proportional to the increasing points. The former is mainly up to the computation amount of (*x* − *x*_0_)(*x* − *x*_1_) ⋯ (*x* − *x*_*n*_) and the derivative operations. Obviously, according to (2), the increase in computational complexity is *O*(*L* ^3^) when the number of extension points *L* increases by 1. But the computational complexity of adaptively increasing collocation points is related to the different wavelet functions. For the wavelet with compact support property such as Daubechies wavelet and Shannon wavelet, the value of *L* is impossible to be infinite. For Haar wavelet which has no smoothness property,* L* can be taken as 0 at most since it need not be extended. For Faber-Schauder wavelet,* L* can be taken as 1 at most. For Daubechies wavelet,* L* can be taken as different values according to the order of vanishing moments, but it must be finite. For the wavelets without compact support property,* L* can take value dynamically, such as Shannon wavelet. The computational complexity of increasing points is mainly up to the wavelet function of itself.

## 3. Construction of the Multilevel Interpolation Operator Based on the Interval Wavelet

Let the definition domain of the image be (*x*_min⁡_, *x*_max⁡_)×(*y*_min⁡_, *y*_max⁡_); the discretization points can be defined as (*x*_*k*_1__^*j*^, *y*_*k*_2__^*j*^), where *j* is a scale parameter and *k*_1_ and *k*_2_ are position parameters. So,

(18) xk1j=xmin⁡+k1xmax⁡−xmin⁡2j,yk1j=ymin⁡+k2ymax⁡−ymin⁡2j,j,k1,k2∈Z.

In addition, *w*_*k*_1_,*k*_2__^*j*(*m*,*n*)^(*x*, *y*) denotes the multiscale wavelet function and the corresponding *m*th and *n*th derivatives with respect to *x* and *y*, respectively. The level set function *ϕ*(*x*, *y*, *t*) and the corresponding derivative function can be discretized as follows:

(19) ϕJ(m,n)(x,y,t) =∑k01=01 ∑k02=01ϕ(xk010,yk020)wk01,k020(m,n)(x,y)  +∑j=0J−1 ∑k11=02j−1 ∑k12=02j−1[αj,k11,k121(t)w2k11+1,2k12j+1(m,n)(x,y)+αj,k11,k122(t)w2k11,2k12+1j+1(m,n)(x,y)+αj,k11,k123(t)×w2k11+1,2k12+1j+1(m,n)(x,y)],

where *j* and *J* are constants, which denote the wavelet scale number and the maximum of the scale number, respectively. *α*_*j*,*k*_11_,*k*_12__^1^, *α*_*j*,*k*_11_,*k*_12__^2^, and *α*_*j*,*k*_11_,*k*_12__^3^ are the wavelet coefficients at the points (*x*_*k*_1__^*j*^, *y*_*k*_2__^*j*^). According to the interpolation wavelet transform theory, the wavelet coefficients can be written as

(20) αj,k1,k21=ϕ(xj+1,2k1+1,yj+1,2k2)−Ijϕ(xj+1,2k1+1,yj+1,2k2),αj,k1,k22=ϕ(xj+1,2k1,yj+1,2k2+1)−Ijϕ(xj+1,2k1,yj+1,2k2+1),αj,k1,k23=ϕ(xj+1,2k1+1,yj+1,2k2+1)−Ijϕ(xj+1,2k1+1,yj+1,2k2+1),

where *I*_*j*_ denotes the multilevel interpolation operator. In order to obtain the multilevel interpolation operator, it is necessary to express the wavelet coefficients *α*_*j*,*k*_1_,*k*_2__^1^, *α*_*j*,*k*_1_,*k*_2__^2^, and  *α*_*j*,*k*_1_,*k*_2__^3^ as a weighted sum of *u* in all of the collocation points in the* J* level. Therefore, we should give the definition of the restriction operator as follows:

(21) Rk1,k2,m1,m2  l,l,j,j={1,xk1l=xm1j,  yk2l=ym2j0,otherwise.

Using the restriction operator, *u*(*x*_2*k*_1_+1_^*j*+1^, *y*_2*k*_2__^*j*+1^),  *u*(*x*_2*k*_1__^*j*+1^, *y*_2*k*_2_+1_^*j*+1^),  and  *u*(*x*_2*k*_1_+1_^*j*+1^, *y*_2*k*_2_+1_^*j*+1^) can be rewritten as

(22) ϕ(x2k1+1j+1,y2k2j+1)=∑n1=02J ∑n2=02JR2k1+1,2k2,n1,n2j+1,j+1,J,Jϕ(xn1J,yn2J),ϕ(x2k1j+1,y2k2+1j+1)=∑n1=02J ∑n2=02JR2k1,2k2+1,n1,n2j+1,j+1,J,Jϕ(xn1J,yn2J),ϕ(x2k1+1j+1,y2k2+1j+1)=∑n1=02J ∑n2=02JR2k1+1,2k2+1,n1,n2j+1,j+1,J,Jϕ(xn1J,yn2J).

Introducing the extension operators* C*1,* C*2, and* C*3, and substituting (22) into (20), the wavelet coefficients can be rewritten as

(23) αj,k1,k21=∑n1=02J ∑n2=02JR2k1+1,2k2,n1,n2j+1,j+1,J,Jϕ(xn1J,yn2J) −[∑n1=02J ∑n2=02J ∑k01=02j0 ∑k02=02j0Rk01,k02,n1,n2j0,j0,J,J×ϕ(xn1J,yn2J)wk01,k02j0(x2k1+1j+1,y2k2j+1)+∑j1=j0j−1 ∑n1=02J ∑n2=02J ∑k11=02j1 ∑k12=02j1(C1k11,k12,n1,n2j1,j1,J,Jw2k11+1,2k12j1+1×(x2k1+1j+1,y2k2j+1)ϕ(xn1J,yn2J)+C2k11,k12,n1,n2j1,j1,J,Jw2k11,2k12+1j1+1×(x2k1+1j+1,y2k2j+1)u(xn1J,yn2J)+C3k11,k12,n1,n2j1,j1,J,Jw2k11+1,2k12+1j1+1×(x2k1+1j+1,y2k2j+1)×ϕ(xn1J,yn2J))]=∑n1=02J ∑n2=02JC1k1,k2,n1,n2j,j,J,Jϕ(xn1J,yn2J).

*α*_*j*,*k*_1_,*k*_2__^2^and *α*_*j*,*k*_1_,*k*_2__^3^ are similar with *α*_*j*,*k*_1_,*k*_2__^1^. From above equation, the extension operator can be obtained as

(24) C1k1,k2,n1,n2j,j,J,J=R2k1+1,2k2,n1,n2j+1,j+1,J,J −[∑k01=02j0 ∑k02=02j0Rk01,k02,n1,n2j0,j0,J,Jϕ(xn1J,yn2J)wk01,k02j0(x2k1+1j+1,y2k2j+1)+∑j1=j0j−1 ∑n2=02J ∑k11=02j1(C1k11,k12,n1,n2j1,j1,J,Jw2k11+1,2k12j1+1(x2k1+1j+1,y2k2j+1)×ϕ(xn1J,yn2J)+C2k11,k12,n1,n2j1,j1,J,Jw2k11,2k12+1j1+1×(x2k1+1j+1,y2k2j+1)ϕ(xn1J,yn2J)+C3k11,k12,n1,n2j1,j1,J,Jw2k11+1,2k12+1j1+1×(x2k1+1j+1,y2k2j+1)ϕ(xn1J,yn2J))].

*C*2 and *C*3 can be obtained with the same method. Therefore, the calculation time complexity of the wavelet transform coefficients *α*_*j*,*k*_11_,*k*_12__^1^, *α*_*j*,*k*_11_,*k*_12__^2^, and *α*_*j*,*k*_11_,*k*_12__^3^ is *O*((1/3)4^2*J*−1^).

Substituting *α*_*j*,*k*_11_,*k*_12__^1^, *α*_*j*,*k*_11_,*k*_12__^2^, and *α*_*j*,*k*_11_,*k*_12__^3^ and* C*1,* C*2, and* C*3 into (2), the multilevel wavelet interpolation operator can be obtained as

(25) In1,n2(x,y) =∑k01=02j0 ∑k02=02j0Rk01,k02,n1,n2j0,j0,J,Jwk01,k02j0(x,y)  +∑j=j0J−1 ∑k1=02j ∑k2=02j(C1k1,k2,n1,n2j,j,J,Jw2k1+1,2k2j+1(x,y)ϕ(xn1J,yn2J)+C2k1,k2,n1,n2j,j,J,Jw2k1,2k2+1j+1×(x,y)ϕ(xn1J,yn2J)+C3k1,k2,n1,n2j,j,J,Jw2k1+1,2k2+1j+1×(x,y)ϕ(xn1J,yn2J)).

Then, (10) can be rewritten as

(26) $$\begin{array}{c} \phi^{J(m,n)}\left(x,y,t\right)=\overset{2^{J}}{\underset{n_{1}}{\sum }}\;\overset{2^{J}}{\underset{n_{2}}{\sum }}I_{n_{1},n_{2}}\left(x,y\right)\phi \left(x_{n_{1}}^{J},y_{n_{2}}^{J}\right). \end{array}$$

Substituting (26) into (13), the multilevel wavelet discretization scheme of Perona-Malik model can be obtained.

The purpose of constructing the multilevel sparse grid approach is to decrease the amount of the collocation points and then improve the efficiency of the algorithm. But the efficiency will be eliminated if the computation complexity of the multilevel wavelet interpolation operator is too high. It is easy to understand that the interpolation wavelet coefficient is the error between the interpolation result and the exact result at the same collocation point. And so, the wavelet coefficient must be the function of the parameter *t*. In other words, the wavelet coefficient should vary with the time parameter *t*. Then, the interpolation operator can be viewed as a nonlinear problem. HPM is an efficient and effective tool to solve nonlinear problem. Aiming to improve the efficiency of the multilevel wavelet interpolation operators, HPM would be employed to construct a novel interpolation operator in this section.

For convenience, *ϕ* and its derivative in (5) should be rewritten as

(27) ∂ϕ∂t=F(t,x,y,ϕ,∂ϕ∂x,∂ϕ∂y,∂2ϕ∂x2,∂2ϕ∂x∂y,∂2ϕ∂y2)(t>0)  ϕ(x,y,0)=ϕ0(x,y),

(28) dϕJ(x,y,t)dt =F[t,x,y,ϕJ(x,y,t),ϕJ(1,0)(x,y,t),ϕJ(0,1)(x,y,t),ϕJ(2,0)(x,y,t),ϕJ(1,1)(x,y,t),ϕJ(0,2)(x,y,t)],

respectively, where

(29) ϕJ(x,y,t) =∑k01=01 ∑k02=01ϕ(xk010,yk020)wk01,k020(x,y)  +∑j=0J−1 ∑k11=02j−1 ∑k12=02j−1[αj,k11,k121w2k11+1,2k12j+1(x,y)+αj,k11,k122w2k11,2k12+1j+1(x,y)+αj,k11,k123w2k11+1,2k12+1j+1(x,y)].

The value of *ϕ* ^*J*^(*x*, *y*, *t*_*n*_) at *t*_*n*_ is denoted by *ϕ*_*n*_, and

(30) F[tn,x,y,ϕJ(x,y,tn),ϕJ(1,0)(x,y,tn),ϕJ(0,1)(x,y,tn),ϕJ(2,0)(x,y,tn),ϕJ(1,1)(x,y,tn),ϕJ(0,2)(x,y,tn)]

is denoted by *F*_*n*_. And then, a linear homotopy function can be constructed as

(31) $$\begin{array}{c} \phi^{J}\left(x,y,t\right)=\left(1-\varepsilon \right)F_{n}+\varepsilon F_{n+1}. \end{array}$$

It is easy to identify the homotopy parameter as

(32) $$\begin{array}{c} \varepsilon \left(t\right)=\frac{t-t_{n}}{t_{n+1}-t_{n}}\;t\in \left[t_{n},t_{n+1}\right]\;\;∴\varepsilon \in \left[0,1\right]. \end{array}$$

According to the perturbation theory, the solution of (31) can be expressed as the power series expansion of *ε*:

(33) $$\begin{array}{c} \phi^{J}=\phi_{0}^{J}+\varepsilon \phi_{1}^{J}+\varepsilon^{2}\phi_{2}^{J}+\cdots . \end{array}$$

Substituting (29) into (27) and rearranging based on powers of *ε*-terms, we have

(34) ε0:    ϕ0J=Fnε1:    ϕ1J=Fn+1−Fn⋮

According to HPM, we obtain the wavelet coefficients *α*_*j*,*k*_1_,*k*_2__^1^(*t*_*n*+1_),  *α*_*j*,*k*_1_,*k*_2__^2^(*t*_*n*+1_), and  *α*_*j*,*k*_1_,*k*_2__^3^(*t*_*n*+1_) at *t*_*n*_ as follows:

(35) αj,k1,k21=ϕ(x2k1+1j+1,y2k2j+1)−Ijϕ(x2k1+1j+1,y2k2j+1)=ϕ(x2k1+1j+1,y2k2j+1) −[∑k01=01 ∑k02=01ϕ(xk010,yk020)wk01,k020(x2k1+1j+1,y2k2j+1)+∑j1=0j−1 ∑k11=02j1 ∑k12=02j1(αj1,k11,k121w2k11+1,2k12j1+1×(x2k1+1j+1,y2k2j+1)+αj1,k11,k122w2k11,2k12+1j1+1×(x2k1+1j+1,y2k2j+1)+αj1,k11,k123w2k11+1,2k12+1j1+1×(x2k1+1j+1,y2k2j+1))],αj,k1,k22=ϕ(x2k1j+1,y2k2+1j+1)−Ijϕ(x2k1j+1,y2k2+1j+1)=ϕ(x2k1j+1,y2k2+1j+1) −[∑k01=01 ∑k02=01ϕ(xk010,yk020)wk01,k020(x2k1j+1,y2k2+1j+1)+∑j1=0j−1 ∑k11=02j1 ∑k12=02j1(αj1,k11,k121w2k11+1,2k12j1+1×(x2k1j+1,y2k2+1j+1)+αj1,k11,k122w2k11,2k12+1j1+1×(x2k1j+1,y2k2+1j+1)+αj1,k11,k123w2k11+1,2k12+1j1+1×(x2k1j+1,y2k2+1j+1))],αj,k1,k23=ϕ(x2k1+1j+1,y2k2+1j+1)−Ijϕ(x2k1+1j+1,y2k2+1j+1)=ϕ(x2k1+1j+1,y2k2+1j+1) −[∑k01=01 ∑k02=01ϕ(xk010,yk020)wk01,k020(x2k1+1j+1,y2k2+1j+1)+∑j1=0j−1 ∑k11=02j1 ∑k12=02j1(αj1,k11,k121w2k11+1,2k12j1+1×(x2k1+1j+1,y2k2+1j+1)+αj1,k11,k122w2k11,2k12+1j1+1×(x2k1+1j+1,y2k2+1j+1)+αj1,k11,k123w2k11+1,2k12+1j1+1×(x2k1+1j+1,y2k2+1j+1))].

Obviously, the calculation time complexity of the wavelet transform coefficients *α*_*j*,*k*_1_,*k*_2__^1^,  *α*_*j*,*k*_1_,*k*_2__^2^, and *α*_*j*,*k*_1_,*k*_2__^3^ is *O*(4^*J*^), which is decreased greatly than in (11) which is *O*((1/3)4^2*J*−1^).

Substituting the wavelet transform coefficient (35) into (29), we obtain

(36) ϕJ(x,y,tn+1) =ϕJ(x,y,tn)  +∆t2[F(tn,x,y,ϕJ(x,y,tn),ϕJ(1,0)(x,y,tn),ϕJ(0,1)(x,y,tn),ϕJ(2,0)(x,y,tn),ϕJ(1,1)(x,y,tn),ϕJ(0,2)(x,y,tn))+F(tn+1,x,y,ϕ0J(x,y,tn+1),ϕ0J(1,0)(x,y,tn+1),ϕ0J(0,1)(x,y,tn+1),ϕ0J(2,0)(x,y,tn+1),ϕ0J(1,1)(x,y,tn+1),ϕ0J(0,2)(x,y,tn+1))].

And the derivative function

(37) ϕJ(m,n)(x,y) =∑k01=01 ∑k02=01ϕ(xk010,yk020)wk01,k020(m,n)(x,y)+∑j=0J−1 ∑k11=02j−1 ∑k12=02j−1[αj,k11,k121w2k11+1,2k12j+1(m,n)(x,y)+αj,k11,k122w2k11,2k12+1j+1(m,n)(x,y)+αj,k11,k123w2k11+1,2k12+1j+1(m,n)(x,y)].

Obviously, the computation complexity is decreased greatly comparing with (26).

## 4. Numerical Experiences and Discussion

In this section, we take some images as examples to illustrate the efficiency of the dynamic interval wavelet interpolation operator based on HPM in partitioning technique on the image processing. In fact, the partitioning technique is a scheme to divide the image into several subimages in the multiscale wavelet numerical method to improve the efficiency. The dynamic interval wavelet provides an adaptive choice scheme for the external collocation points to eliminate the boundary effect of the subimages. Perona-Malik equation is employed as the denoising model, which is an anisotropic diffusion image denoising model that was proposed by Perona and Malik. It has been widely used in various image processing fields. It can be represented as the nonlinear partial differential equations:

(38) ∂u(x,y,t)∂t=div⁡(c(|∇u|)∇u),u(x,y,0)=f(x,y),

where (*x*, *y*) denotes pixel position, *t* is the time parameter, *f*(*x*, *y*) is the 2D image being processed, *u*(*x*, *y*, *t*) is the image after diffusion processing, and *u*(*x*, *y*, 0) is the initial value. div⁡ denotes the divergence operator, ∇*u* denotes the gradient operator, and *c*(|∇*u*|) denotes the diffusion coefficient, which is nonnegative decreasing function of the image gradient modulus. It is usually taken as

(39) $$\begin{array}{c} c\left(\left|\nabla u\right|\right)=\frac{1}{1+{\left(\nabla u/k\right)}^{2}} \end{array}$$

or

(40) $$\begin{array}{c} c\left(\left|\nabla u\right|\right)=\exp\left[-{\left(\frac{\nabla u}{k}\right)}^{2}\right], \end{array}$$

where *k* is a constant.

**Figure 1:**
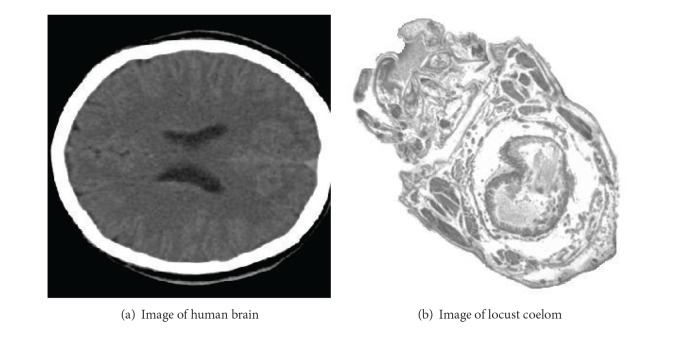
Original images.

Two different medical images are taken as examples to test the characteristic of different interpolation wavelets, which is showed in Figure 1. One is the human brain (Figure 1(a)), which has so clear contour that that image cannot be represented as a continuous function near the contour. The Gibbs phenomenon is possible to be introduced into the image near the boundary. So, this can be used to test the advantages of the multiscale wavelet approximation comparing with the difference operator. Another one is the image of the locust coelom, which has many microgrooves without clear boundary. This image is used to test the characteristic of different interpolation wavelets, which is showed in Figure 1(b).

### 4.1. Comparison between the Sparse Grid Approach and the Finite Different Method

**Figure 2:**
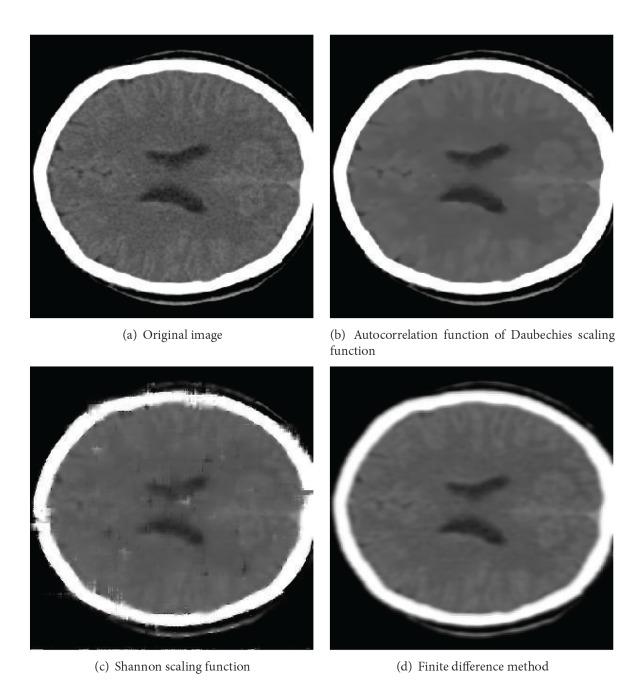
Comparison between different numerical methods for image denoising (time step *τ* = 0.00001, terminal time *t* = 0.00005).

It has been mentioned above that the brain image is used to test the difference between the sparse grid approach and the finite difference method and the difference between different wavelet functions which are taken as the basis functions in the sparse grid approach. In this experiment, all the results are obtained by solving the Perona-Malik equation with different methods, which have been showed in Figure 2.

Two interpolation wavelet scaling functions are employed to test the dynamic sparse grid approach for image denoising proposed in this paper. The Shannon wavelet possesses the smoothness and or the orthogonality but has no compact support property. Daubechies scaling function possess almost all the excellent properties in numerical algorithm such as smoothness, orthogonality, and compact support property. But what we utilized in this research is the autocorrelative function of the Daubechies scaling function, which keeps the better edge preserving property although it loses the orthogonality. It can be easily observed from Figure 2(c) that the evident artifacts appeared in the denoised image obtained by the Shannon sparse grid approach. That is, the Gibbs phenomenon has appeared in the Shannon scaling function representation of the image near the boundary. In contrast to the Shannon wavelet, the denoised image (Figure 2(b)) obtained by the Daubechies wavelet sparse grid approach has clear boundary. It is easy to understand that the compact support property of the wavelet scaling function is helpful to eliminate the Gibbs phenomenon and so to improve the numerical performances of the wavelet numerical methods.

Comparing with the sparse grid approaches, the finite difference method utilizes the difference operator to approximate the derivative in Perona-Malik equation, which decreases the value of the derivative to some extent. Therefore, the edge of the brain contour is smoothed in denoised images; this is showed in Figure 2(d). It should be noticed that the edge of the denoised image obtained by the Shannon wavelet sparse grid approach is more clear than that obtained by the finite difference method, in despite of the appearing artifacts.

### 4.2. Comparison between the Dynamic Interval Wavelet and the Static Interval Wavelet

For convenience of comparison, we call the interval interpolation wavelet constructed by the Lagrange interpolator as static interval wavelet and the interval interpolation wavelet based on the Newton interpolator as the dynamic interval wavelet. The difference between two interval wavelets above is the choice of the parameter *L*. The value of *L* is constant to the static interval wavelet, and it varies with both of the boundary condition and the condition number of the system ODEs obtained from the sparse grid approach.

**Figure 3:**
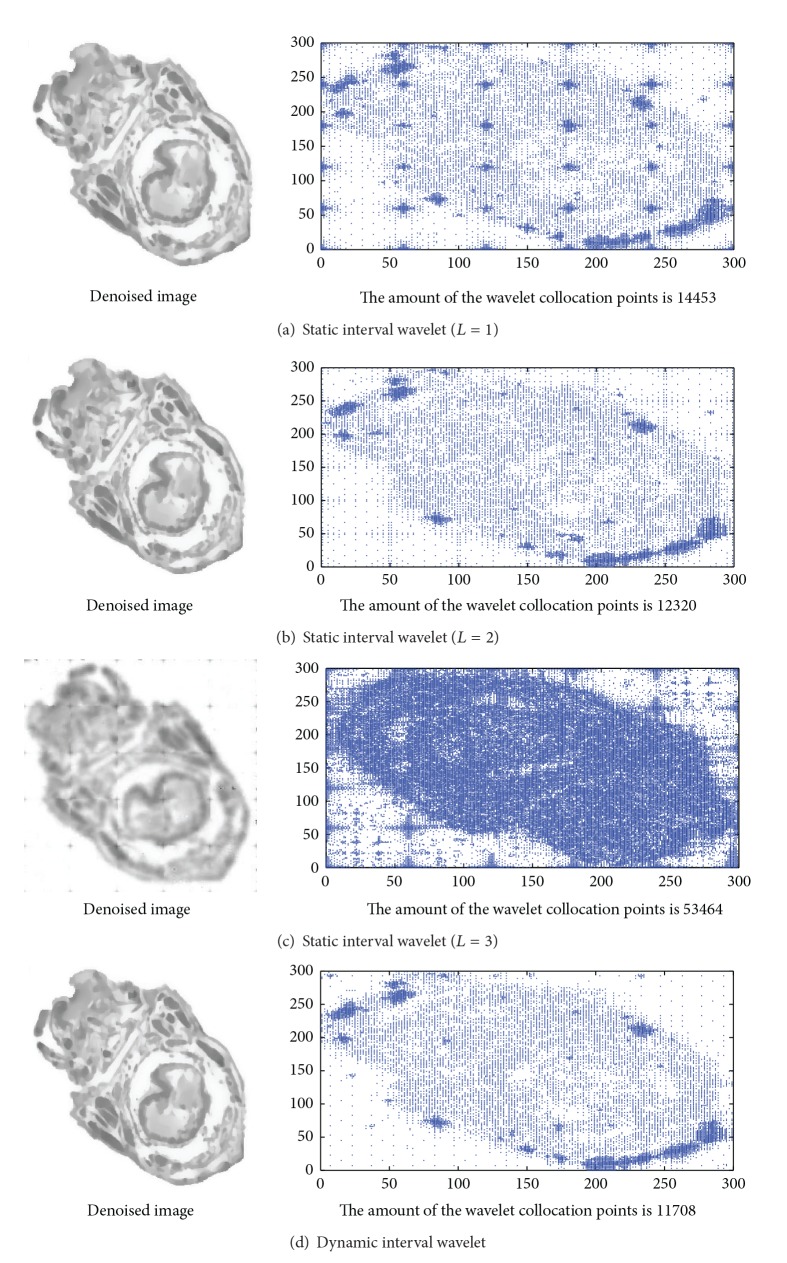
Comparison between the dynamic interval wavelet and the static interval wavelet.

The purpose of constructing of the interval wavelet is to eliminate the boundary effect in the partitioning technique on the image denoising process. In this section, the image of locust coelom (300 ∗ 300 pixels) is taken as example to compare the difference between the dynamic and static interval wavelets. According to the partitioning technique, the image is divided evenly into 25 parts for simplification. So, the size of each image block is 60 ∗ 60 pixels (Figure 3). According to the sparse grid approach based on HPM, the calculation amount decreases from (300 ∗ 300)^3^ to 25 ∗ (60 ∗ 60)^3^. It has been mentioned that there are many ways to eliminate the boundary effect such as the extension method and the interval wavelet method. There is no doubt that the interval wavelet method is more efficient than the extension method. According to the interval interpolation wavelet based on the Lagrange interpolator, the amount of the external collocation points *L* is a constant. With increase of *L*, the calculation amount will increase correspondingly.

*L* is taken as 1, 2, and 3, respectively, in the experiments. It is easy to be observed from Figures 3(a)–3(c) that there are more collocation points near the boundary of each of block images in all 3 cases. In fact, the adaptive increase of the collocation points can also eliminate the boundary effect. Therefore, there are no artifacts appearing in the denoised images in the first two cases. But the increase of the collocation points can increase the calculation amount greatly. According to the definition of the interval interpolation wavelet based on the Lagrange operator, the increase of *L* can improve the smoothness and the precision of the approximated function near the boundary. This is helpful to decrease the boundary effect in theory. In contrast to the theory, the collocation points in the whole image domain increased so much that the artifacts appeared in the denoised subimages when *L* = 3 comparing to other two cases (Figure 3(c)). As a matter of fact, this is caused by the increase of the condition number of the system of ODEs obtained by the sparse grid approach. That is, the increase of the value of *L* can induce the condition number change greatly; this is showed in Table 1. It has been pointed out in Section 2 that if the condition number cond(*A*) = 10^*k*^, then you may lose up to *k* digits of accuracy on top of what would be lost to the numerical method due to loss of precision from arithmetic methods. This also illustrates that the condition number must be taken into account in the dynamic interval wavelet.  Figure 3(d) is the result obtained by the dynamic interval sparse grid approach. The distribution of the collocation points in Figure 3(d) is just correlative with the image content itself and is not correlative with the partitioning scheme of the image anymore. The amount of the wavelet collocation points also decreased accordingly.

### 4.3. Adaptability of the Wavelet Collocation Points

**Figure 4:**
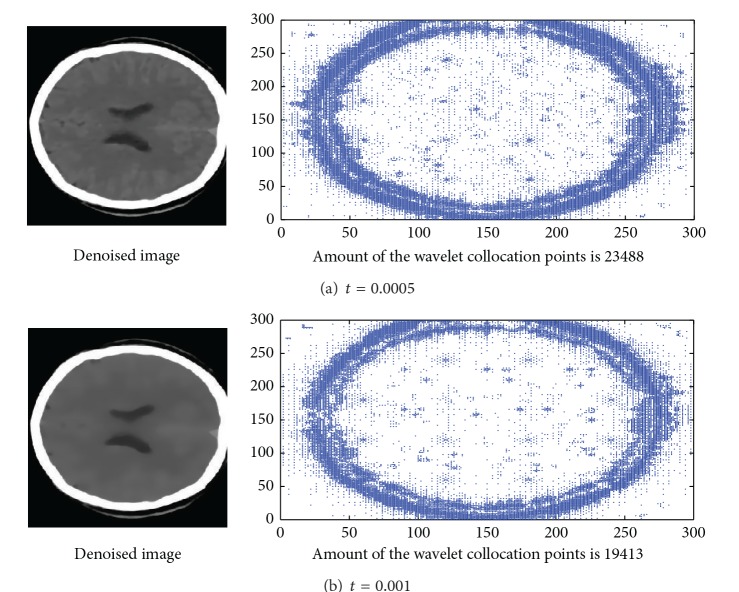
Adaptability of the multiscale sparse grid approach in image denoising (time step *τ* = 0.00001).

In this research, the dynamic interpolation operator was viewed as a nonlinear problem, and so HPM is employed in construction of the dynamic interpolation wavelet defined in interval. This is helpful to improve the efficiency of the multilevel wavelet interpolation operators. In this section, the autocorrelation function of the Daubechies scaling function is employed to construct the dynamic interval wavelet. The brain image is taken as example to test the precision and efficiency of the HPM-based dynamic interval wavelet proposed in this research. The experiment results were showed in Figure 4. It is easy to be observed that the noise pixels of the brain images were smoothed completely and the edges of the brain contour were preserved perfectly. With the increase of the iteration times, more and more trivial objects such as the noise pixels are being smoothed, and more areas in the brain image are becoming smoother. Accordingly, the amount of the wavelet collocation points should be smaller and smaller. This has been illustrated in Figures 4(a) and 4(b). In this experiment, the time step *τ* = 0.00001; the definition domain of the parameter *t* is [0, 0.001]. The experiment results show that the amount of the wavelet collocation points decreases from 23488 to 19413 with the parameter *t* increasing from 0.0005 to 0.001. According to the finite difference method, the amount of the collocation points should be 90000, which is greater than the sparse grid approach, evidently. This illustrates that the dynamic interval sparse grid approach proposed in this paper is more efficient than the finite difference method.

## 5. Conclusions

The dynamic interval wavelet and the corresponding numerical method proposed in this paper are intrinsically an adaptive choice scheme on the external collocation points. In partitioning technique about the image processing, the dynamic sparse grid approach can be used to eliminate the boundary effect and improve the algorithm efficiency. In this method, the wavelet interpolation operator is constructed based on the homotopy perturbation method, which can decrease the calculation amount greatly. In addition, comparing with the finite difference method, the dynamic interval sparse grid approach can preserve the object edge more clear, especially in the case that the edge is sharper. For simplification, the image is divided evenly into several parts according to the partitioning scheme in the experiments. It is obvious that the partitioning scheme can be adaptive, which can improve the efficiency furthermore.

## Figures and Tables

**Table 1:** Condition number of each image block at different times.

Block number	Condition number				
	*t* = 0.00001	*t* = 0.00002	*t* = 0.00003	*t* = 0.00004	*t* = 0.00005
1	9.6829	7.7193	6.7935	6.8257	5.9730
2	13.7539	12.8756	11.8760	11.8703	11.3319
3	9.2841	8.8832	8.1376	7.8737	6.8713
4	9.6829	7.7193	6.7935	6.8257	5.9730
5	1.6816	1.6931	1.7153	1.7378	1.8923
6	13.9357	12.9657	11.8891	11.8757	10.3356
7	13.7543	12.8757	11.8765	10.8701	9.3318
8	13.7556	12.8781	11.8776	11.8734	11.3329
9	43.9354	32.9663	31.8882	28.8749	21.3346
10	3.2389	2.9137	2.2917	1.9365	1.2919
11	8.5692	6.9416	6.6971	5.2875	4.3907
12	31.9823	29.3266	28.8330	25.6736	20.1976
13	15.1617	14.7818	13.8967	12.5738	12.3428
14	12.9614	12.1973	10.9887	10.1725	9.3189
15	4.6593	3.7938	2.5476	2.3789	1.9916
16	14.6835	13.9864	13.1838	12.8897	11.3156
17	12.8113	12.1031	10.1763	9.5627	8.2474
18	14.9834	13.6523	12.7719	12.1658	11.7829
19	13.6689	12.1791	11.1782	10.5977	8.9664
20	125.4782	110.3379	98.9073	89.7761	80.2749
21	1.6816	1.6931	1.7153	1.7378	1.8923
22	2.4589	2.6623	3.7662	3.8955	4.5811
23	43.2983	39.6744	36.7943	32.1079	28.6179
24	211.5877	198.7219	180.7089	86.9125	81.8510
25	185.7428	170.5897	160.0987	52.1757	83.0421
